# Maternal vitamin D supplementation during pregnancy and lactation to promote infant growth in Dhaka, Bangladesh (MDIG trial): study protocol for a randomized controlled trial

**DOI:** 10.1186/s13063-015-0825-8

**Published:** 2015-07-14

**Authors:** Daniel E. Roth, Alison D. Gernand, Shaun K. Morris, Brendon Pezzack, M. Munirul Islam, Michelle C. Dimitris, Shaila S. Shanta, Stanley H. Zlotkin, Andrew R. Willan, Tahmeed Ahmed, Prakesh S. Shah, Kellie E. Murphy, Rosanna Weksberg, Sanaa Choufani, Rashed Shah, Abdullah Al Mahmud

**Affiliations:** Department of Paediatrics, University of Toronto and the Centre for Global Child Health, Hospital for Sick Children, 686 Bay Street, Toronto, ON Canada; Child Health Evaluative Sciences, SickKids Research Institute, Hospital for Sick Children, 686 Bay Street, Toronto, ON Canada; Department of Nutritional Sciences, Penn State University, 110 Chandlee Laboratory, University Park, PA USA; Centre for Nutrition and Food Security, International Centre for Diarrhoeal Disease Research (ICDDR,B), 68 Shaheed Tajuddin Ahmed Sarani, Mohakhali, Dhaka, 1212 Bangladesh; Division of Neonatology, Mt. Sinai Hospital, 600 University Avenue, Toronto, ON Canada; Department of Obstetrics and Gynecology, University of Toronto and Mt. Sinai Hospital, 600 University Avenue, Toronto, ON Canada; Genetics and Genome Biology, SickKids Research Institute, Hospital for Sick Children, 686 Bay Street, Toronto, ON Canada; Department of Health and Nutrition, Save the Children USA, 2000 L Street NW, Suite 500, Washington, DC USA; Centre for Child and Adolescent Health, International Centre for Diarrhoeal Disease Research (ICDDR,B), 68 Shaheed Tajuddin Ahmed Sarani, Mohakhali, Dhaka, 1212 Bangladesh

**Keywords:** Bangladesh, Lactation, Length, Linear growth, Nutrition, Pregnancy, Stunting, Vitamin D

## Abstract

**Background:**

Vitamin D regulates bone mineral metabolism and skeletal development. Some observational studies have suggested that prenatal vitamin D deficiency increases the risk of adverse pregnancy and/or birth outcomes; however, there is scant evidence from controlled trials, leading the World Health Organization to advise against routine vitamin D supplementation in pregnancy. Importantly, little is known about the effect of maternal vitamin D status on infant linear growth in communities in South Asia where stunting is highly prevalent and maternal-infant vitamin D status is commonly suboptimal.

**Methods/Design:**

The Maternal Vitamin D for Infant Growth study is a randomized, placebo-controlled, dose-ranging trial of maternal vitamin D supplementation during pregnancy and lactation in Dhaka, Bangladesh. The primary aims are to estimate (1) the effect of maternal prenatal oral vitamin D_3_ supplementation (4200 IU/wk, 16,800 IU/wk, or 28,000 IU/wk, administered as weekly doses) versus placebo on infant length at 1 year of age and (2) the effect of maternal postpartum oral vitamin D_3_ supplementation (28,000 IU/wk) versus placebo on length at 1 year of age among infants born to women who received vitamin D 28,000 IU/wk during pregnancy. Generally healthy pregnant women (n = 1300) in the second trimester (17–24 weeks of gestation) are randomized to one of five parallel arms: placebo 4200 IU/wk, 16,800 IU/wk, or 28,000 IU/wk in the prenatal period and placebo in the postpartum period or 28,000 IU/wk in the prenatal period and 28,000 IU/wk in the postpartum period. Household- and clinic-based follow-up of mother-infant pairs is conducted weekly by trained personnel until 26 weeks postpartum and every 3 months thereafter. The primary trial outcome measure is length for age z-score at 1 year of age. Anthropometric measurements, clinical information, and biological specimens collected at scheduled intervals will enable the assessment of a range of maternal, perinatal, and infant outcomes.

**Discussion:**

The role of vitamin D in maternal and infant health remains unresolved. This trial is expected to contribute unique insights into the effects of improving maternal-infant vitamin D status in a low-income setting where stunting and adverse perinatal outcomes represent significant public health burdens.

**Trial registration:**

ClinicalTrials.gov identifier: NCT01924013. Registered on 13 August 2013

**Electronic supplementary material:**

The online version of this article (doi:10.1186/s13063-015-0825-8) contains supplementary material, which is available to authorized users.

## Background

Many low-income countries in Asia have experienced substantial recent reductions in mortality rates among children younger than 5 years of age [1]; for example, Bangladesh has met United Nations Millennium Development Goal 4—a two-thirds reduction in child mortality between 1990 and 2015. However, childhood undernutrition remains a persistent global public health problem [2], particularly in Sub-Saharan Africa and South Asia [3]. In Bangladesh, stunting (height or length less than 2 standard deviations below the standard median for age and sex) was estimated to affect 51 % of children younger than 5 years of age in 2004, but declined to 43 % in 2007 and to 41 % in 2011 according to national surveys [4].

Fetal and early childhood growth is believed to have a critical influence on early childhood infectious disease susceptibility, mortality, and long-term functional and social outcomes, particularly during the first 1000 days of pre- and postnatal development [5, 6]. Postnatal linear growth faltering—an abnormally slow velocity of length/height gain that may eventually lead to stunting—often begins early in infancy in low-income settings (i.e., within the first 3 months of life [7]), suggesting an important contribution by prenatal determinants, including epigenetic and endocrine factors that regulate growth in the first months of life. However, the causal pathways implicated in early childhood stunting remain poorly understood [8, 9].

Vitamin D is a well-established regulator of bone mineral metabolism and skeletal development [10]. Children with severe vitamin D deficiency rickets have secondary hyperparathyroidism and demineralization of the growing skeleton, as well as impairment of bone elongation [11]. Vitamin D treatment of affected children leads to an acceleration of linear growth accompanied by resolution of the hyperparathyroidism [11–13]. Effects of improvements in vitamin D status on linear growth in infants and children without rickets have not been widely studied; however, in one trial in India, weekly vitamin D supplementation of low birth weight infants reduced the risk of stunting in adjusted analyses [14]. Vitamin D status is therefore a logical candidate as a modifiable nutritional determinant of linear growth in communities where stunting is prevalent and vitamin D status is commonly suboptimal. In South Asia, there is a high prevalence of biochemical vitamin D deficiency among women and young infants [15]; in Dhaka, we found that 34 % of pregnant women at 26–29 weeks gestation (*N* = 160) had serum 25-hydroxyvitamin D (25(OH)D) concentrations less than 30 nmol/L, and that 64 % had 25(OH)D levels less than 50 nmol/L [16], a threshold for sufficiency adopted by the Institute of Medicine (IOM) [17]. In the absence of maternal vitamin D supplementation, postnatal infant 25(OH)D concentration was low in the neonatal period, but gradually increased over the first several months of life (unpublished observations). Therefore, in Bangladesh, we have observed that the period of greatest vulnerability with respect to perinatal vitamin D deficiency overlaps with a critical period of fetal and early infant growth. Because maternal vitamin D status is the predominant determinant of fetal and neonatal vitamin D stores [18], prenatal vitamin D supplementation represents the optimal approach to testing the effect of improving maternal-infant vitamin D status on fetal-infant growth and other health outcomes.

Observational studies of the association between maternal prenatal vitamin D status and infant anthropometry have yielded conflicting findings [19–23]. The effects of prenatal vitamin D supplementation on infant length have not been widely studied in controlled trials. In a study conducted in the 1970s in London, England, prenatal vitamin D supplementation reduced the risk of small for gestational age (SGA) versus control, but there was no significant effect on birth length [24]; however, the infants in the vitamin D group had significantly greater mean length at 1 year of age [25]. We recently completed a randomized, placebo-controlled, double-blinded trial of maternal third-trimester vitamin D_3_ supplementation in Dhaka, in which 160 women were randomized to receive either 35,000 IU/wk or placebo until delivery. Maternal mean 25(OH)D was significantly higher at delivery after receiving vitamin D versus placebo (134 vs. 39 nmol/L; *P* < 0.001; *N* = 133). There was a parallel difference in cord 25(OH)D (103 nmol/L in vitamin D group vs. 39 nmol/L in placebo group; *P* < 0.001; *N* = 132) [16]. Analyses of the effect of prenatal vitamin D supplementation on infant length among 134 infants followed up to 1 year of age revealed that infants born to women in the vitamin D group had mean length for age z-scores (LAZs) at 1 year that were significantly greater than infants in the placebo group (mean = 0.44 z-score units higher; 95% confidence interval [CI], 0.06–0.82) [26]. This corresponded to an approximate halving of the prevalence of stunting (11 % vs. 21 %), which would constitute a substantial public health benefit. The effect appeared to be attributable to a significantly accelerated increase in mean LAZ from birth to 1 month of age (change of 0.53 z-score in vitamin D versus 0.19 z-score in placebo group; *P* = 0.004) rather than a significant difference in birth length [26]. However, some animal [27] and observational [21, 28] studies have suggested an effect of maternal prenatal vitamin D status on growth of the fetus.

There has been considerable recent interest in the role of vitamin D in maternal and infant health outcomes in low-income settings [29]. Some observational studies have suggested that antenatal vitamin D deficiency increases the risk of adverse pregnancy and birth outcomes, including SGA [30, 31]. However, there is scant evidence from controlled clinical trials [31, 32], and the World Health Organization (WHO) recently advised against routine vitamin D supplementation in pregnancy until further data become available [33]. Building on our earlier findings of a beneficial effect of prenatal vitamin D supplementation on infant length in a preliminary trial [26], we are conducting a larger dose-ranging trial of maternal vitamin D_3_ supplementation in Dhaka, Bangladesh. The primary aim is to investigate the effect of maternal vitamin D supplementation on infant length at 1 year of age, but the trial also provides an opportunity to study a range of other potential vitamin D–responsive outcomes.

## Methods/Design

The Maternal Vitamin D for Infant Growth (MDIG) trial is randomized, placebo-controlled, dose-ranging trial of maternal vitamin D supplementation during pregnancy and lactation to improve infant linear growth in Dhaka, Bangladesh (ClinicalTrials.gov identifier NCT01924013). A detailed trial protocol was developed in accordance with the SPIRIT guidelines [34] through a collaboration between the International Centre for Diarrhoeal Disease Research (ICDDR,B; Dhaka, Bangladesh) and the Hospital for Sick Children (Toronto, ON, Canada) and with funding from the Bill and Melinda Gates Foundation through its Achieving Healthy Growth platform (http://gcgh.grandchallenges.org/grantopportunities/pages/healthygrowth.aspx).

The first participant was enrolled on 18 March 2014; enrollment is expected to proceed until August 2015; and planned data collection will continue until 2018.

### Objectives

The primary aims of the trial are to estimate (1) the effect of maternal prenatal oral vitamin _D3_ supplementation (4200 IU/wk, 16,800 IU/wk, or 28,000 IU/wk administered as weekly doses) versus placebo on infant length at 1 year of age in Dhaka, Bangladesh; and (2) the effect of maternal postpartum oral vitamin D_3_ supplementation (28,000 IU/wk) versus placebo on length at 1 year of age among infants born to women who received vitamin D 28,000 IU/wk during pregnancy. Secondary anthropometric outcomes include birth size and the prevalence of SGA, the prevalence of stunting at 1 year of age, and mean attained LAZ and stunting prevalence at 2 years of age.

Clinical, biochemical, and microbiological surveillance of enrolled mother-infant pairs will enable a range of secondary objectives to be addressed, including (1) estimation of the effect of maternal vitamin D supplementation on the incidence of acute respiratory infections (ARIs) during early infancy; (2) exploration of the roles of specific hormones, nutrients, environmental contaminants, and inflammatory markers in the mediation or modification of the effect of vitamin D on fetal and infant growth, with particular emphasis on the role of the parathyroid hormone (PTH) system; and (3) investigation of the role of epigenetic modification of genes involved in perinatal vitamin D metabolism in infant stunting.

### Setting and participants

Recruitment, enrollment, and clinical activities are based at the Maternal and Child Health Training Institute (MCHTI), a Bangladeshi national government-operated facility that provides health care to pregnant women and children in its referral area in central Dhaka, Bangladesh. MCHTI has outpatient antenatal care clinics, a labor and delivery unit, and inpatient pediatric services. The Dhaka wards and/or unions near MCHTI that are included in the trial area include Kamrangirchar, Azimpur, Lalbag, and Hazaribag. About three-fourths of the participants are expected to be residents of Kamrangirchar, a group of urban slums on the Buriganga River along the periphery of Dhaka city. The area is densely populated, with a total population of about 300,000, of whom approximately 265,000 reside in slum settlements [35]. The literacy rate is approximately 29 % (compared with the national average of 32 %) and more than 30 % of the residents have monthly incomes of 5000 taka (about US$60) or less. Income earners are mainly day laborers (e.g., rickshaw pullers), and many men work in local tanneries or operate small businesses. However, the socioeconomic status of residents varies, owing to the presence of some universities and government offices in the area.

The MDIG trial is an efficacy study designed to test a specific biological mechanism implicated in fetal and infant growth. We therefore established a detailed set of eligibility criteria that will optimize the internal validity of the trial by minimizing the number of withdrawals due to loss to follow-up or serious adverse events, acknowledging that this may theoretically reduce the generalizability of the findings. Among women presenting to MCHTI for antenatal care, participant eligibility is based on the following inclusion criteria: age 18 years and older, intention to reside in the trial area for at least 18 months, and currently at 17–24 completed weeks of gestation (i.e., 17 weeks + 0 days to 24 weeks + 0 days, inclusive) based on recalled last menstrual period (LMP) and ultrasound performed by MCHTI technicians (Just Vision 400, Toshiba, Tokyo, Japan; SonoAce X8, Samsung Medison, South Korea). All potential participants undergo routine ultrasound. If there is a difference of more than 10 days between gestational age (GA) calculated using the recalled LMP and second-trimester ultrasound, the estimated date of delivery is adjusted as per the second-trimester ultrasound. Otherwise, the GA date based on recalled LMP is used [36]. If more than one ultrasound report is available, GA estimation is based on the earliest of the ultrasounds. If the earliest ultrasound was performed in the first trimester and there is a difference of more than 5 days between GA calculated using the recalled LMP and first-trimester ultrasound, the estimated date of delivery is adjusted as per the first-trimester ultrasound [36].

Pregnant women are not enrolled if any of the following exclusion criteria apply: self-reported history of any medical condition or medications that may predispose to vitamin D sensitivity, altered vitamin D metabolism, and/or hypercalcemia (e.g., active tuberculosis or current therapy for tuberculosis, sarcoidosis, history of renal and/or ureteral stones, parathyroid disease, renal or liver failure, or current use of antiseizure medications); high-risk pregnancy based on point-of-care (POC) testing (hemoglobin [Hb] <70 g/L; proteinuria, defined as ≥300 mg/dl based on urine dipstick; hypertension, defined as systolic blood pressure ≥140 mmHg and/or diastolic blood pressure ≥90 mmHg); high-risk pregnancy based on maternal report and/or past ultrasound findings (multiple gestation, major congenital anomaly, severe oligohydramnios); unwillingness to stop taking non-study vitamin D or calcium supplements or a multivitamins containing calcium and/or vitamin D; currently prescribed vitamin D supplements as part of a physician’s treatment plan for vitamin D deficiency; and/or previous enrollment in the MDIG trial during a prior pregnancy.

All participants provide written informed consent before randomization. The informed consent process is undertaken in conjunction with the iterative eligibility assessment: preliminary screening (based on age, location of residence, plans to remain in referral area), detailed medical screening by a study physician, POC testing (blood pressure, urine dip, Hb), and obstetric ultrasound. Women may choose to exit the enrollment process at any time. Among prospective participants who complete the screening process and are found to meet all criteria, a detailed discussion of the trial is undertaken before written informed consent is sought. Eligible pregnant women are encouraged to discuss trial participation with their spouse and/or other family members before finalizing consent. Two additional consent procedures are undertaken at a later time for supplemental study procedures: (1) husbands of enrolled participants are approached to obtain consent for paternal anthropometry and blood specimen collection, and (2) enrolled participants are approached at or beyond 30 weeks of gestation to request the participation of their infant in an ARI substudy during the 0- to 6-month postnatal period (detailed methods of the ARI substudy will be presented elsewhere).

### Design and sample size

**Fig. 1:**
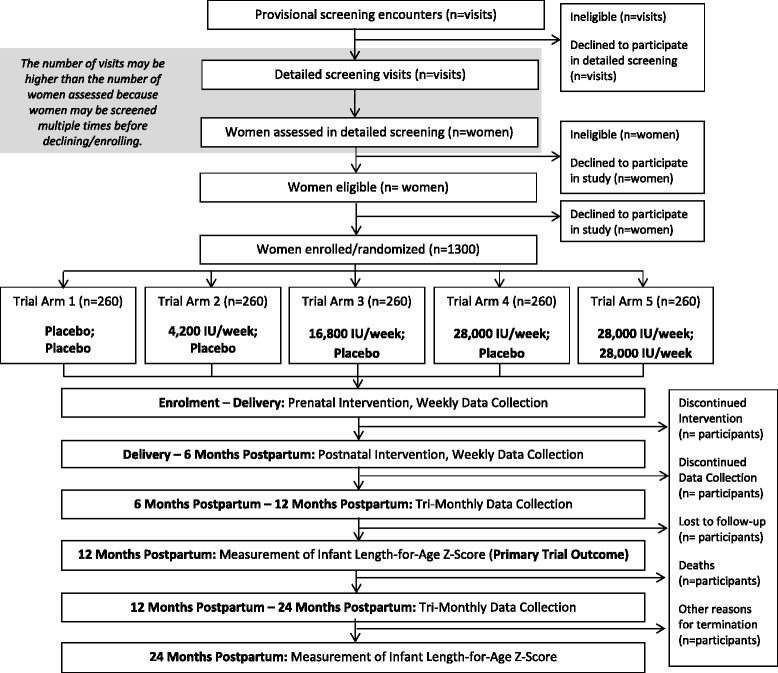
Maternal Vitamin D for Infant Growth (MDIG) trial flow diagram. The allocated intervention in each group is shown as “prenatal intervention; postpartum intervention”

The MDIG trial is a randomized, placebo-controlled, double-blinded, dose-ranging trial with a 1:1 allocation ratio across five parallel groups (Fig. 1). A total of 1300 pregnant women will be randomized, with a target of allocating 260 women in each group (Fig. 1). The primary outcome analysis will be a between-group comparison of mean LAZs at 1 year of age, assuming up to 15 % attrition from each group before this time point. To assess the effect of prenatal vitamin D, we plan to perform five primary between-group analyses, comprising each vitamin D dose versus placebo (three comparisons) and comparisons between adjacent doses (two comparisons). A conservative approach to addressing multiple testing is to partition the α (risk of type I error) among the five comparisons (conventional overall 0.05 divided by 5); thus, each between-group comparison will be tested as a two-sided test with an α of 0.01, assuming 90 % power. With 220 analyzable participants per group (about 85 % of total enrolled), the minimum detectable difference in LAZ will be 0.40 z-score units, which equates to approximately 1.0 cm at 1 year of age based on WHO growth standards. In our preliminary trial, we observed an increase in LAZ of 0.44 at 1 year attributable to prenatal vitamin D supplementation, which corresponded to an increase of 1.1 cm (95 % CI, 0.06–2.0), adjusted for sex [26].

The secondary analysis at 2 years of age will enable detection of an approximately 1.2-cm difference between groups. For the postpartum effect analysis of 28,000 IU/wk versus placebo postpartum among women who received 28,000 IU/wk antenatally, the comparison at 1 year of age will have a minimum detectable difference of 0.31 z-score units (assuming 90 % power, a 5 % risk of a type I error for a two-sided test, and at least 220 participants per group). Based on WHO growth standards, this is approximately 0.77 cm at 1 year of age and 0.96 cm at 2 years of age. Because the α is not subject to multiple testing for the postpartum effect analysis, there is greater precision to detect a smaller difference compared with the prenatal effect analysis. In order to enroll 1300 participants, we expect to provisionally screen approximately 15,000 pregnant women for eligibility. The major reasons for exclusion during preliminary screening are GA beyond 24 weeks and residence outside the study area.

### Randomization and allocation concealment

The allocation sequence was generated by the trial statistician (ARW) using a computer-generated random number sequence according to a simple randomization scheme (i.e., no stratification or blocking) [36]. The list was provided to the Toronto Institute for Pharmaceutical Technology (TIPT), the pharmaceutical company that has produced and packaged the supplements in individual participant supplement packs, each of which is labeled with a unique participant identifier. The prelabeled supplement packets containing both prenatal and postpartum supplements (in separate vials) are sequentially allocated to participants according to their order of enrollment. Participants, investigators, field personnel, study laboratory staff, and data analysts are blinded to vitamin D or placebo group allocation. The allocation scheme will be made available to the Data and Safety Monitoring Board (DSMB) in cases in which individual participants need to be unmasked because of suspected supplement-related adverse events (i.e., hypercalcemia or clinical features suggestive of vitamin D toxicity).

### Interventions and control

#### Vitamin D doses

The primary objective of the MDIG trial is to establish evidence of a causal relationship between maternal-infant vitamin D status and infant stature. A randomized dose-ranging trial enables robust causal inferences based on dose–response effects, in addition to relatively unbiased comparisons with the placebo group. Three vitamin D_3_ doses (4200 IU/wk, 16,800 IU/wk, and 28,000 IU/wk) were selected on the basis of preliminary data, published literature, and consideration of the 2010 IOM dietary reference intakes [38] (see Additional file 1 for detailed rationale). Pharmacological principles and empirical data indicate that weekly doses of vitamin D achieve 25(OH)D levels similar to equivalent daily doses [39]; thus, the equivalent daily doses in increasing order are 600 IU/day, 2400 IU/day, and 4000 IU/day. Our previous pharmacokinetic findings among pregnant women in Dhaka [16, 40] have generally been consistent with published estimates of the vitamin D-to-25(OH)D dose–response relationship in non-pregnant adults (i.e., an approximately 0.7 nmol/L increase in 25(OH)D at steady state for each 1 μg/day of vitamin D_3_ [41, 42]). The preliminary pharmacokinetic studies have provided evidence of the biochemical efficacy, tolerability, and short-term safety of doses of up to 35,000 IU/wk in pregnancy in the target population. Hollis et al. previously reported that doses up to 4000 IU/day were well tolerated and efficacious at raising 25(OH)D in pregnant women in the United States [43].

#### Study tablets

Oral vitamin D_3_ (cholecalciferol) is provided as small tablets (10 mm diameter) that are custom-manufactured by TIPT in Toronto, ON, Canada (www.tipt.com). Each participant’s weekly dose consists of a single tablet. Across trial groups, the tablets vary only by the vitamin D_3_ dose but are identical with respect to appearance, taste and excipients (Table 1). Vitamin D_3_ content per tablet has been verified in each production batch (Table 2), with a very high degree of tablet uniformity (F. Martinuzzi, personal communication, December 2014).

**Table 1:** Vitamin D_3_ and excipient content of custom-made tablets for the Maternal Vitamin D for Infant Growth trial^a^

Content type	Tablet contents
Granules^b^	Vitamin D_3_^c^
	α-Tocopherol^d^
	Corn starch
	Medium-chain triglycerides
	Acacia gum
	Sucrose
	Dibasic calcium phosphate
Additional tablet excipients	Croscarmellose sodium
	Silicon dioxide
Tablet coating	Hydroxypropylmethylcellulose
	Polyethylene glycol 6000
	Titanium dioxide

^a^Composition information provided by the manufacturer (Toronto Institute of Pharmaceutical Technology, Toronto, ON, Canada)

^b^Placebo granules were identical except for the absence of vitamin D_3_

^c^Dry vitamin D_3_ 100 GFP/HP (BASF, Mississauga, ON, Canada)

^d^α-Tocopherol is included as an antioxidant to stabilize the vitamin D_3_. The vitamin E content of each tablet is 2.8 mg/wk (about 0.4 mg/day), which is far below recommended dietary allowances for pregnancy (15 mg/day) and lactation (19 mg/day)

**Table 2:** Vitamin D_3_ content of custom-made trial tablets, based on postproduction coated tablet content analysis by high-performance liquid chromatography^a^

Label claim	Cholecalciferol content (% label claim)		
	Batch 1^b^ (November 2013)	Batch 2 (April 2014)	Batch 3 (June 2014)
4200 IU	106 %	103 %	101 %
16,800 IU	105 %	101 %	102 %
28,000 IU	102 %	101 %	99 %

^a^Modified United States Pharmacopeia method, as reported by the manufacturer (Toronto Institute of Pharmaceutical Technology, Toronto, ON, Canada). Results shown reflect the batches completed and assayed at the time of manuscript preparation, not the full set of batches to be used in the trial.

^b^Similar results obtained in testing by independent third-party laboratory (Alpha Laboratories, Toronto, ON, Canada)

#### Supplement administration

Supplementation begins at enrollment (second trimester) and continues on a weekly basis throughout pregnancy and the postpartum period until 26 weeks postpartum (6 months). Study personnel maintain and store all tablet supplies in locked study offices and directly observe tablet ingestion during home or clinic visits. However, for periods of up to 4 weeks during which the participant plans to travel or during holidays, advance doses may be given to the participant to take unobserved but with telephone reminders. Tablets are swallowed whole with any liquid (e.g., water, juice, tea) or, if necessary, chewed with or without a small amount of soft food. Tablets may be consumed with or without food, which has a negligible impact on vitamin D absorption [44]. Although scheduled for specific 7-day intervals, a missed dose may be administered as a late dose on any day up to 7 days after the scheduled date of administration; thereafter, subsequent dosing continues as originally scheduled, even if this incurs at an interval of less than 7 days between the late dose and the subsequent regularly scheduled dose. If a participant refuses a dose or complains of feeling unwell (e.g., nausea) on the scheduled day of dose administration, the dose may be deferred for up to 7 days. If a participant vomits during or within 20 minutes of dose administration (observed by study personnel), the dose may be repeated immediately or may be deferred to another day (within 7 days). If vomiting occurs more than 20 minutes after a dose was swallowed, the dose is considered successfully administered. If a participant refuses doses for 3 consecutive weeks and in the third week expresses the intention to continue avoidance and/or refusal of the dose, weekly supplementation may be discontinued, but clinical follow-up and scheduled data collection procedures may continue.

#### Micronutrient cointerventions

Calcium 500 mg/day as calcium carbonate (Calbo; Square Pharmaceuticals, Dhaka, Bangladesh) and iron and folic acid (66 mg elemental iron per day, and 350 μg folic acid per day included in the standard formulation available in Bangladesh) are provided to all participants throughout the intervention phase (prenatal period and up to 6 months postpartum). Although prenatal calcium supplementation is not formally recommended or widely practiced in Bangladesh, standard provision of calcium was incorporated into the trial design to mitigate any rate-limiting effects of serious maternal dietary calcium deficits on fetal growth or other outcomes. From a practical standpoint, the investigation of vitamin D supplementation in the context of routine calcium supplementation will facilitate the translation of trial findings into anticipated future contexts in which supplemental calcium is more routinely provided. We considered the recent recommendation by the WHO advising 1.5–2 g/day of supplemental calcium for pregnant women with low dietary calcium intake [45], but this policy has not been widely adopted, owing to substantial logistical and technical limitations. Supplemental calcium and/or vitamin D not prescribed by the study protocol are prohibited during the intervention phase of the trial. Participants are questioned on a weekly basis as to whether they have consumed other nutrient supplements. Participants who continue to consume non-study supplements after an initial warning are no longer permitted to continue receiving the allocated intervention, although clinical follow-up and scheduled data collection procedures may continue.

### Visit schedule

Following enrollment and randomization of pregnant women at 17–24 weeks of gestation, weekly supplementation (vitamin D or placebo) occurs throughout the prenatal period and during the first 6 months postpartum. Data collection continues during the non-intervention phase beginning at 26 weeks postpartum. The primary anthropometric outcome will be ascertained at 1 year of age, and follow-up of infants will continue until 2 years of age. The prenatal timeline of study activities is structured in terms of GA (in weeks), based on the recalled LMP and/or ultrasound results. Postnatal follow-up visits are scheduled on the basis of the infant’s age in weeks (rather than anniversaries of the birthdate); however, the timing of major visits is operationally described in terms of months or years for simplicity (e.g., 3 months = 13 weeks, 6 months = 26 weeks, 9 months = 39 weeks, 1 year = 12 months = 52 weeks, and 2 years = 24 months = 104 weeks).

Participant visits are scheduled on a weekly basis during the prenatal and 6-month postpartum intervention phase. The majority of visits are conducted in the participants’ homes; however, selected visits that involve specimen collection or anthropometry are preferentially conducted in a clinic at MCHTI. Participants are strongly encouraged to seek a facility-based delivery at MCHTI; however, perinatal data and specimen collection are attempted when feasible for home deliveries or for deliveries that occur at facilities other than MCHTI. Beyond 6 months, clinic-based visits occur at 3-month intervals until 2 years of age (Additional file 2). Participant retention is promoted through frequent interaction between field-level study personnel and the participants (weekly during the intervention phase). Adherence to the schedule of clinic visits is facilitated by compensation of participants for costs of transportation and time away from the home and/or work.

All study activities (e.g., supplementation, visits, maternal and infant specimen collection) cease for a mother-infant pair when any one of the following events occurs: (1) the 24-month infant visit is completed, (2) participant (maternal) death before delivery, (3) stillbirth or infant/child death, (4) consent for all types of follow-up is withdrawn, or (5) loss to follow-up. Loss to follow-up is considered to have occurred if (1) study staff determine conclusively that the participant cannot be contacted for the purposes of data collection for the duration of the period of scheduled follow-up (e.g., they have been informed of the participant’s emigration from Bangladesh) or (2) at least 3 months have passed since the scheduled but missed 24-month postnatal visit. A participant may be absent for a period of time (and thus miss supplement dosing, specimen collection, and other data collection) and yet return to follow-up without being excluded from the study.

### Data collection procedures

Trained personnel collect participant data using questionnaires, POC clinical tests, abstraction of prenatal ultrasound reports, anthropometric measurements, and specimen collection throughout the interventional and observational phases of the study (Additional file 2). Data are recorded using a series of standardized data collection forms that are customized for each visit type. To overcome low literacy in the population, field personnel administer all questionnaires as structured oral interviews. A wide range of lifestyle and health-related items (e.g., obstetric history, tobacco use, sun exposure) are included in baseline and follow-up questionnaires. Dietary intake of vitamin D, calcium, phosphorus, and phytates are assessed among participants (mothers) at enrollment and 6 months postpartum using a customized, focused food frequency questionnaire. Infant dietary patterns are assessed at weekly visits to assess breastfeeding patterns and timing of introduction and frequency of consumption of specific complementary foods. A household survey to assess socioeconomic status is conducted at the first scheduled home visit 1 week after enrollment, at the 9-month home postnatal visit, and at the 21-month postnatal home visit.

POC tests include blood pressure measurement, urine dipstick testing, and Hb concentration estimation. Maternal systolic and diastolic blood pressure is measured using an automated digital blood pressure monitor (WatchBP Home; MicroLife USA, Clearwater, FL, USA). Two readings are taken at least 1 minute apart and recorded; if either diastolic or systolic measurements differ between the paired readings by more than 10 mmHg, a third measurement will be performed. For analysis, paired readings will be averaged; where a third reading has been obtained, the single discordant reading will be excluded. Urine dipsticks (Urinalysis Reagent Test Strips; Siemens Diagnostics, Erlangen, Germany) are used at enrollment as part of the eligibility assessment and then after enrollment if high blood pressure is detected (systolic blood pressure ≥140 mmHg and/or diastolic blood pressure ≥90 mmHg) or if there are symptoms (e.g., dysuria) suggestive of urinary tract infection (to test for leukocyte esterase). Maternal Hb will be measured at enrollment (as part of the eligibility screening process) in a finger-stick blood sample using a handheld hemoglobinometer (Hb 201; HemoCue, Ängelholm, Sweden). Infant Hb is measured using the same HemoCue device at 6 months of age.

Standardized procedures for infant length, weight, and head circumference (HC) measurements were adapted from the INTERGROWTH-21st study standard operating procedures [46]. Other published resources were adapted to develop standard protocols for measurement of upper arm length (UAL) [47], mid-upper arm circumference (MUAC) [48], and rump-to-knee length (RKL) [49]. Infants are measured independently by two study personnel at each visit. The paired measurements are compared, and if they differ by more than the threshold values (7 mm for length; 5 mm for HC, UAL, MUAC, and RKL; and 50 g for weight), a second set of measurements is performed. If the second pair of measurements differs by more than the threshold values, the procedure is repeated a third time (except for infant weight, which is measured using a digital scale and thus expected to be concordant). Length, weight, and HC are measured at birth, at one randomly assigned weekly visit during the first 2 months after birth, and then every 3 months starting at the 3-month visit. MUAC, UAL, and RKL are measured at 3, 6, 12, and 24 months; UAL and RKL are also measured at birth.

Length, HC, MUAC, UAL, and RKL are recorded to the last completed unit. For each infant at each time point, the mean of acceptable paired measures will be used in primary analyses. Infant weight is measured to the nearest 5 g (up to 10 kg) and to the nearest 10 g (for >10 kg) using a digital infant scale (seca 334; Seca, Hamburg, Germany). Length is measured to the last completed 0.1 cm (1 mm) using a wooden length board (Infant/Child ShorrBoard; Weigh and Measure, Olney, MD, USA). Initially, we selected a length board with a counter display and a ball bearing mounted sliding footboard (Harpenden infantometer; Holtain, Crymych, UK), but decalibration of the counter occurred frequently, particularly when the instrument was moved among assessment locations. Therefore, as of 15 December 2014, the ShorrBoard was adopted because of its easier portability and robustness. HC, MUAC, and UAL are measured using a flexible tape measure, and RKL is measured using a rigid caliper (Holtain-Kahn Abdominal Caliper; Holtain). These dimensions are measured to the last completed 0.1 cm (1 mm). An anthropometric standardization workshop was conducted before launch of anthropometric data collection, which was adapted from the methods of the WHO [50] and the INTERGROWTH-21st study group [51].

Maternal and paternal weight and height are measured at MCHTI according to standard methods adapted from the *National Health and Nutrition Examination Survey Anthropometry Procedures Manual* of the Centers for Disease Control and Prevention [47], using a digital floor scale (HD-318; Tanita, Tokyo, Japan) and a stadiometer (Leicester Height Measure device; Chasmors, London, UK). Measurements are performed in duplicate. Third measurements are taken if the following discrepancies are noted between the paired measures: more than 0.5 kg for weight and more than 2 cm for height.

### Specimen collection

#### Blood specimen collection

Trained phlebotomists collect maternal blood, paternal blood, cord venous and arterial blood, and infant blood specimens according to standard sampling procedures (Table 3). Following filling, BD Vacutainer serum (red top) and ethylenediaminetetraacetic acid (EDTA) (lavender or blue top) blood collection tubes (BD Diagnostics, Franklin Lakes, NJ, USA) are inverted according to the manufacturer’s instructions and centrifuged immediately (EDTA) or after 30 minutes (serum) at low speed for 15 minutes, and the supernatant (plasma or serum) is transferred via micropipettes in 0.25-ml aliquots into prelabeled microfuge tubes (Fisherbrand Free-Standing Microcentrifuge Tubes with Screw Caps; Thermo Fisher Scientific, Waltham, MA, USA). Whole blood aliquots (1.5 or 0.5 ml, depending on size of blood draw and laboratory requirements) are drawn from EDTA tubes immediately after mixing (before centrifugation) for paternal blood, maternal blood at delivery, and cord venous blood specimens. Within 30 minutes of blood collection, serum and plasma aliquots are placed in a portable ultracold freezer (Shuttle ULT-25N; Stirling Ultracold, Athens, OH, USA) and maintained at less than −70 °C. At the completion of each workday, frozen aliquots are transported in the portable freezer or a liquid nitrogen canister to an upright −80 °C freezer maintained with backup generator support and constant monitoring. One serum aliquot from each maternal and infant 3- and 6-month blood specimen is reserved at 2–8 °C for same- or next-day serum calcium measurement.

**Table 3:** Biological specimen collection schedule and planned laboratory analyses in the Maternal Vitamin D for Infant Growth (MDIG) trial in Dhaka, Bangladesh

		Maternal-fetal						Infant			Father
		Baseline	30 wk gestation	Delivery	Cord blood	3 mo postpartum	6 mo postpartum	3 mo	6 mo	12 and 24 mo	
Blood/serum/plasma	Calcium	R^a^	R	R	R	R	R	R	R		
	Phosphate, alkaline phosphatase, creatinine								S^a^		
	25(OH)D	R		S	S		S	S	S		
	1,25(OH)_2_D, whole/bioactive PTH(1–84), C-terminal PTH(73–84), intact PTH, FGF-23	S		S	S			S	S		
	PTHrP	S		S	S	S	S	S	S		
	IGF-1, IGFBP-1, IGFBP-3				S				S		
	Ferritin								S		
	Retinol, folate, cadmium			S							
	C-reactive protein, IL-6, IL-8, TNF-α			S	S						
	Epigenetic studies			S	S						S
	Collection for future analyses	R	R	R	R	R	R	R	R	R	R
Placenta	Epigenetic studies			R							
Urine	Calcium:creatinine ratio			R					S		
	Calcium, phosphate, fluoride, cyclic AMP								S		
	Collection for future analyses	R		R					R		
Breast milk	PTHrP					S		S			
	Collection for future analyses					R		R			
Nasal swab^b^	PCR for respiratory viruses							R^b^	R^b^		
	PCR for *Streptococcus* carriage density							R^b^	R^b^		

*FGF* fibroblast growth factor, *IGF* insulin-like growth factor, *IGFBP* insulin-like growth factor-binding protein, *IL* interleukin; *PTHrP* parathyroid hormone–related protein, *R* routine, *S* subset, *TNF* tumor necrosis factor

^a^Routine analyses to be performed in all specimens collected; subset analyses to be performed in a sub-set of stored specimen aliquots

^b^Nasal swabbing is performed at any time from 1 wk to 6 mo of age if criteria for acute respiratory infection are met

#### Cord blood collection

The technique and timing of umbilical cord clamping and cutting is determined by the attending physician or birthing attendant (i.e., cord clamping time is not set by the study protocol, but is recorded). As soon as possible within 30 minutes of delivery of the placenta, a site on the umbilical cord attached to the placenta is cleansed using dry cotton gauze to wipe away any maternal blood. The umbilical vein is cannulated, and blood is collected into collection tubes in the following order: (1) serum (red top) tube, (2) EDTA (lavender top) tube, and (3) trace element (blue top) tube with EDTA. Any remaining extractable blood is collected in EDTA (lavender top) tubes. Attempts are made to collect a total of 20–40 ml of cord blood. If feasible, the umbilical artery is cannulated and a serum (red top) tube also filled.

#### Placental weight and specimen collection

After collection of cord blood, the umbilical cord and membranes are removed from the placenta; the blood is drained; and the placenta is weighed to the nearest 0.5 g (iBALANCE i2500; My Weigh Canada, Vancouver, BC, Canada). Placental specimens from at least two quadrants of the placenta are collected and divided into three specimen types: (1) full disc biopsies of approximately 0.5-cm thickness that are stored in 10 % formalin at room temperature for histopathological examination; (2) small tissue samples (approximately 0.2 cm^3^) from the fetal surface of the placenta that are stored in RNA Save (Biological Industries Israel Beit Haemek, Kibbutz Beit Haemek, Israel) at 2–8 °C for 48 h and then transferred to −80 °C until epigenetic and/or gene expression studies are done; and (3) small tissue samples (about 0.2 cm^3^) from the maternal surface of the placenta that are stored and used similarly to the specimens from the fetal surface. Specimens from each quadrant are collected and pooled to create aliquots that represent the entire placental disc (one pooled histopathology sample and multiple pooled RNA Save samples from each of the fetal and maternal sides).

#### Urine and breast milk specimen collection

Maternal urine samples are self-collected into sterile plastic containers at baseline after enrollment (but before first supplement dose), during labor before delivery, and at the 6-month postpartum visit. For each sample, 1.5-ml aliquots are stored at −80 °C for future analyses. In addition, an aliquot from the intrapartum sample is held at 2–8 °C for same- or next-day analysis of the calcium:creatinine (Ca:Cr) ratio (see below). Infant urine samples are collected at the 6-month postnatal visit using infant urine collection bags that adhere to the perineum; 1.5-ml aliquots are stored at −80 °C for future analyses. At 3 and 6 months postpartum, hand-expressed midfeed breast milk samples are collected and stored at −80 °C for future analyses. Between birth and 6 months of age, midturbinate nasal swabs for viral PCR will be collected from infants who meet clinical criteria for upper or lower respiratory tract infections. (Detailed methods for the ARI substudy will be presented elsewhere.)

#### Laboratory analyses

Vitamin D status is based on the serum 25(OH)D concentration [52] assessed by high-performance liquid chromatography tandem mass spectrometry performed at the Analytical Facility for Bioactive Molecules (Hospital for Sick Children, Toronto, ON, Canada). This assay captures 25(OH)D_2_ and 25(OH)D_3_, and enables separate quantification of the C3-epi-25(OH)D moiety. The current approach uses an Agilent 1290 Infinity high-performance liquid chromatography system (Agilent Technologies, Mississauga, ON, Canada) interfaced with an AB Sciex QTRAP 5500 mass spectrometer (SCIEX, Concord, ON, Canada), and separation by a Kinetex 2.6-μm pentafluorophenyl 100mm×2.1mm column (Phenomenex, Torrance, CA, USA); however, iterative improvements to the assay system will ensure it remains state-of-the-art throughout the trial. In current use, average intraassay percent coefficient of variation (CV%) is 4 % and average interassay CV% is 6 %. External quality control mechanisms include participation in the Vitamin D External Quality Assessment Scheme (DEQAS, http://www.deqas.org/; London, UK) and use of the National Institute of Standards and Technology (Gaithersburg, MD, USA) standard reference material.

Serum calcium is measured by colorimetric measurement of calcium–Arsenazo complex (Olympus-Beckman Coulter autoanalyzer system; Beckman Coulter, Brea, CA, USA) in the clinical biochemistry laboratory at ICDDR,B (Dhaka, Bangladesh). Maternal and infant serum calcium is routinely monitored in all participants throughout the intervention phase of the trial (Table 3). To address specific mechanistic hypotheses, several other biochemical analytes will be measured in serum or plasma aliquots and urine samples from a subset of participants (Table 3). Details of these substudies will be described elsewhere. For epigenetic studies, DNA and mRNA will be extracted from blood and placental specimens using techniques previously described [53]. Genomic DNA will be subjected to bisulfite modification, and amplified PCR products will be analyzed by quantitative pyrosequencing to assess DNA methylation at several consecutive CpG sites within an approximately 150-bp region at the promoters of two genes involved in vitamin D metabolism (*CYP27B1* and *CYP24A1*). Where possible, paternal whole blood specimens will be collected to enable the identification of allele-specific patterns. Gene expression (mRNA) from placental specimens (fetal tissue) will be assessed by real-time PCR. Epigenetic analyses will be performed at the Weksberg laboratory at the Hospital for Sick Children (Toronto, ON, Canada). Molecular microbiological analyses of nasal specimens will be performed at the virology and microbiology laboratories at ICDDR,B. (Details of the ARI substudy will be described elsewhere.)

### Data management

A site supervisor reviews all data collection forms for completeness and protocol deviations and/or violations before sending them to the data management center at ICDDR,B on a weekly basis. Electronic (scanned) versions of all forms are also regularly saved for long-term storage. The database was designed using SQL Server 2008 and data are entered using Visual Studio 2010 (both from Microsoft, Redmond, WA, USA). A set of range and consistency checks are built into the data capture system to provide immediate feedback to data entry personnel regarding errors or inconsistent data. Double data entry is used to further reduce the rate of date entry errors.

### Outcome measures

The primary trial outcome measure is LAZ at 1 year of age, where *length* refers to an infant’s fully extended crown-to-heel distance in the supine position. Growth faltering in resource-poor settings primarily occurs early [7], and any effects of the intervention are expected to be apparent by 1 year of age. Our preliminary trial data revealed a discernible effect of prenatal vitamin D supplementation on infant LAZ at 1 year [26]. Because some infants might not be reached at exactly 1 year of age (52 weeks), measurements taken up to 60 weeks will be included in the primary 1-year outcome analysis, implying a range of 364–420 days. LAZ based on WHO growth standards will be used to account for sex imbalances between groups and variance in exact age at the time of measurement. Infant follow-up will continue to 2 years of age (with continued treatment masking) to establish the persistence of effects, to capture potential catch-up growth, and because stunting prevalence does not stabilize until 18–24 months of age [7]. To improve uniformity with respect to the timing of the 2-year visit, it will be scheduled at 24 months of age (104 weeks) but may be completed up to 27 months of age.

LAZs and z-scores for weight for age (WAZ), weight for length (WFLZ), and head circumference for age (HCAZ) will be calculated according to the sex-specific WHO growth standards (WHO-GS) [54, 55] using the STATA igrowup package (http://www.who.int/childgrowth/software/en/). Extreme z-scores will be flagged by the WHO Anthro software (less than −6 SD or greater than 6 SD for LAZ, greater than 5 or less than −6 for WAZ, greater than 5 or less than −5 for HCAZ, and greater than −5 or less than 5 for WFLZ). These extreme values will be manually reviewed to ensure they are not the result of data recording or entry errors. Real values that are extreme low outlying z-scores are expected to be contributed primarily by infants who had early preterm births and of very low birth weight. Other secondary growth outcomes (UAL, MUAC, and RKL) and postnatal growth velocity will be analyzed using raw values or age- and sex-standardized z-scores based on WHO standards (except for RKL).

### Statistical analysis

For the primary trial efficacy outcome analysis, mean LAZ (at 1 year of age) will be compared across groups using analysis of variance. To assess the effect of prenatal vitamin D on mean LAZ at 1 year of age, we plan to perform five primary between-group analyses: each prenatal vitamin D dose versus placebo (three pairwise comparisons), as well as comparisons between all adjacent vitamin D doses (two pairwise comparisons). Because the primary hypotheses relate to differences between two groups, each analysis will be akin to an independent-samples *t* test. The primary effect measure for each comparison will be expressed as a mean difference between groups with 95 % CI. However, an overall α for statistical significance for all five comparisons will be 0.05 (two-sided), and the Holm test will be used to account for multiple testing. Therefore, even if the 95 % CI for a particular mean difference does not include zero, it is possible that it may not be considered statistically significant when all five comparisons are presented together. The postpartum effect analysis will be the comparison of mean LAZ at 1 year of age between infants of mothers who received 28,000 IU/wk versus placebo in the postpartum period (among women who received 28,000 IU/wk prenatally), using a statistical approach similar to that described for the prenatal effect analysis. The nominal αfor statistical significance will be 0.05 (two-sided test) for this analysis. Because there is only one primary pairwise comparison related to the postpartum effect, no adjustment for the multiplicity of outcomes is planned.

In the primary analyses, an intention-to-treat (ITT) approach will be used, meaning participants will be considered exactly as randomized, regardless of supplementation adherence or completeness of follow-up and without adjustment for baseline covariates, and also without imputation for missing data; that is, infants for whom LAZ is unavailable at 1 year of age will be excluded from the analysis. However, because the trial is aimed at establishing a biological mechanism of effect, per-protocol analyses will also be performed by restricting to participants who consumed at least 90 % of all scheduled doses. Conventional ITT analyses of postnatal growth outcomes typically disregard between-individual variations in GA at birth; however, because the WHO-GS was based on a full-term cohort [54], this may lead to differential misclassification of preterm infants with respect to LAZ if length is plotted on the growth chart using the chronological age. Although we plan to follow the convention in the primary analysis, we will perform a sensitivity analysis in which LAZ will be assigned to preterm infants using the age corrected for GA at birth (rather than chronological age). Several other sensitivity analyses are planned. Multivariable adjustment will be used for covariates that substantially differ across groups at baseline. A missingness analysis will be performed to understand the pattern of missing data (in particular to detect differential loss of data across groups), and multiple imputation methods will be used in sensitivity analyses to correct for these losses. Subgroup and interaction analyses will be based on the following covariates: GA at birth, infant sex, maternal baseline vitamin D status, maternal height, and maternal supplement adherence.

Two secondary approaches will be used to examine the effects of vitamin D on linear growth. Regression spline models, with knots at the major scheduled follow-up visit time points, will be used to analyze changes in mean LAZ over discrete time intervals. Interaction terms for time and group allocation will be used to test between-group differences in the changes in LAZ during discrete time intervals. As well, participants will be classified as “stunted” if LAZ is less than −2, and both cross-sectional and longitudinal comparisons will be undertaken to assess the relative risks of stunting across groups. In longitudinal analyses of LAZ or stunting described above, generalized estimating equations with robust variance estimation will be employed to account for within-subject correlation of repeated measures [56].

Summary measures (e.g., means, frequencies, proportions, incidence rates) and effect estimates (i.e., regression coefficients) will be reported as point estimates and 95 % confidence intervals. In general, risk ratios with corresponding CIs will be used to compare dichotomous outcomes across trial groups (e.g., prevalence of SGA), and differences in means will be used for between-group comparisons of continuous variables. *P* values will be reported to three decimal places, with *P* values less than 0.001 reported as less than 0.001. Statistical analyses will be performed using the STATA software package (StataCorp, College Station, TX, USA) with analyses of primary trial outcomes performed in a blinded fashion. Plans of analysis to address secondary objectives will be presented elsewhere.

### Participant safety monitoring

Participant safety during the intervention phase is monitored by study personnel based on (1) a checklist of maternal symptoms that may indicate vitamin D toxicity or other medical concerns during weekly follow-up visits, (2) passive and active monitoring for maternal and infant clinical events, and (3) maternal serum calcium measurement at scheduled intervals during the intervention phase (baseline, 30 weeks of gestation, delivery, 3 months postpartum, and 6 months postpartum). Secondary safety measures include infant serum calcium measurement at 3 and 6 months of age and maternal urinary calcium:creatinine at the time of delivery.

Serum calcium is the best available biomarker of vitamin D toxicity [57, 58]; therefore, hypercalcemia is the primary biochemical safety outcome. Nonetheless, on the basis of our own experience with doses equivalent to up to 5000 IU/day (see above) and previously published trials conducted in the United States using doses up to 4000 IU/day during pregnancy [43] and up to 6400 IU/day during lactation [59], we do not anticipate any episodes of true hypercalcemia or other supplement-related adverse events.

*Possible hypercalcemia* is defined as a single serum calcium concentration above 2.60 mmol/L. This threshold is based on a conservative upper limit of the normal range that accommodates expected variation in serum calcium throughout pregnancy [60], but it avoids the unnecessary operational complexity of using a normal range that varies by stage of gestation. Any participant with possible hypercalcemia detected through scheduled blood sampling (or at the time of a clinical serious adverse event; e.g., hospitalization) will be asked to provide a second sample within 24 h of the time that the study physician receives a report of an abnormal value. Abnormal serum calcium results will always be reported and confirmed before the time of the following week’s supplement dose (to enable a decision to be made about withholding supplementation). Severe derangements in serum calcium will be managed as urgently as possible, including referral for hospitalization.

*Confirmed hypercalcemia* is defined as serum calcium concentration above 2.60 mmol/L in two consecutive specimens and will be considered the primary supplement-related adverse event. The second, confirmatory specimen is required owing to the possibility of a laboratory error, but clinical management will not await the second assay if the participant is symptomatic. If the repeat serum calcium is normal, then supplementation will continue and a further repeat serum calcium will be measured 1 week after the first abnormal result to confirm that it remains within the normal range. In the unlikely event that a participant has confirmed hypercalcemia, study personnel will follow the clinical course until normalization of serum calcium or 1 month postpartum (whichever occurs later). Participants with mild and asymptomatic hypercalcemia may not require referral or treatment beyond the cessation of supplementation and will continue to participate in study follow-up. Participants with confirmed or suspected moderate to severe hypercalcemia (serum Ca >2.80 mmol/L and/or symptomatic) will be assessed at a hospital by a clinician with expertise in the treatment of hypercalcemia.

In previous prenatal vitamin D supplementation trials in Dhaka, we used regular monitoring of maternal urinary Ca:Cr ratio as a screening measure for vitamin D toxicity [16, 40]. However, this test is non-specific and frequently led to the need for repeat testing that was burdensome but uninformative; in no cases did an isolated Ca:Cr ratio indicate vitamin D toxicity. Therefore, regular urine Ca:Cr ratio assessment is not employed as a primary clinical safety monitoring tool in the present trial. However, we will measure urinary Ca:Cr ratio in mothers at delivery as a screening test for hypercalciuria rather than for overt vitamin D toxicity. Abnormal values (Ca:Cr ratio >1 mmol/mmol) will prompt repeat testing, and either of the following criteria will be considered a presumptive diagnosis of hypercalciuria: two consecutive urine samples with Ca:Cr ratio above 1 mmol/mmol or one urine sample with Ca:Cr ratio above 1 mmol/mmol in the presence of persistent symptoms suggestive of possible uro- and/or nephrolithiasis. Participants with persistent symptoms of renal colic or hypercalciuria will be referred for renal ultrasound to assess for the presence of urolithiasis or nephrolithiasis. Participants with uro- or nephrolithiasis will be referred for consideration by the DSMB for a decision regarding unblinding and possible discontinuation of the study supplement, which is to be decided on a case-by-case basis. Biochemical evidence of hypercalciuria alone will not trigger urgent DSMB review, but it will be reviewed at regular intervals. However, participants with hypercalciuria and an absence of stones will undergo repeat urine Ca:Cr ratio assessment 1 month after initial diagnosis. If hypercalciuria is persistent, a repeat ultrasound will be undertaken.

When caregivers permit scheduled infant blood sampling, serum calcium will be routinely measured. The normal range of infant serum calcium is higher than in adults, and normal values may be as high as 3.05 mmol/L [61–66]. However, because there are few reference data for infants specifically at 3 and 6 months, we have chosen to use a more conservative threshold of greater than 2.80 mmol/L to define above-range infant values at the 3- and 6-month visits, which will prompt repeat assessment.

Clinical events will be monitored and documented by study physicians, and serious adverse events will include all deaths and hospitalizations (with the exception of admissions to the hospital for uncomplicated vaginal or cesarean delivery). The only clinical events that will routinely lead to discontinuation of study supplementation are those adverse events that are ascertained to be supplement-related or the diagnosis of a medical condition that potentially or theoretically increases sensitivity to vitamin D supplementation following clinical review by the Trial Steering Committee (TSC): confirmed hypercalcemia (see definition above), symptomatic vitamin D deficiency diagnosed by a study physician or consultant physician (e.g., osteomalacia), fetal or infant death, or onset of a medical condition or initiation of a medication following enrollment that may reasonably predispose to vitamin D sensitivity, altered vitamin D metabolism, and/or hypercalcemia (e.g., tuberculosis or therapy for tuberculosis, sarcoidosis, renal and/or ureteral stones, parathyroid disease, renal or liver failure, or use of anticonvulsants). The occurrence of a serious adverse event, in of itself, will not automatically lead to study supplementation cessation.

Research ethics committee/institutional review board approval was obtained from the Hospital for Sick Children Research Ethics Board (REB1000039072) and the ICDDR,B Ethical Review Committee (ERC; PR number 13055). A DSMB was created by the ERC of ICDDR,B. An external international member of the TSC reviews reports submitted to the DSMB and reports to the TSC. There are no formal interim analyses of efficacy or stopping rules planned for this trial.

## Discussion

Since the publication of the 2010 IOM report on vitamin D and the design of the MDIG trial and dosing regimens in 2012, there have been several new reports of biochemical and clinical findings of prenatal vitamin D supplementation trials conducted in Iran [67–69], the United Arab Emirates [70], Pakistan [71], New Zealand [72], and Australia [73]. However, in none of these studies did researchers report postnatal infant growth outcomes, and all trials enrolled fewer than 100 participants per arm, suggesting they lacked power to detect effects on most pregnancy-related and infant clinical outcomes. The results of a relatively larger trial (*N* = 549) recently completed in rural Pakistan (NCT01229189) have not yet been released publicly. There are at least three other large trials underway: the Maternal Vitamin D Osteoporosis Study, for which the primary outcome is neonatal whole body bone mineral content [74]; the Vitamin D Antenatal Asthma Reduction Trial [75]; and the Vitamin D Supplementation during Pregnancy for Prevention of Asthma in Childhood (ABCvitaminD trial; ClinicalTrials.gov identifier NCT00856947). However, these and other registered prenatal vitamin D trials are being conducted in high-income countries.

The MDIG trial will therefore offer unique insights into the effect of improving maternal-infant vitamin D status in a low-income setting where vitamin D insufficiency is common and there is a high prevalence of infant linear growth faltering. The trial is primarily designed and powered to evaluate the dose–response effects of vitamin D on infant length, an outcome that is directly aligned with the classical role of vitamin D in mineral metabolism and bone health but may also be interpreted as a surrogate marker for organ (including brain) growth and development. Serial specimen collection and frequent clinical follow-up will enable a range of secondary objectives to be addressed, including tests of mechanistic hypotheses (e.g., mediation of the beneficial vitamin D supplement effect on skeletal length by PTH suppression) and assessments of the effects of the intervention on infant morbidity (in particular, the incidence of acute respiratory infections in the first 6 months after birth). Longer-term surveillance of the infant cohort will ideally enable the assessment of other important clinical outcomes (e.g., asthma, neurodevelopment) that may be influenced by the non-classical role of vitamin D in immunoregulation and extraskeletal cellular proliferation and differentiation.

## Trial status

As of July 2015, enrollment is ongoing.

## Additional files

Additional file 1:
**Summary of the rationale for the maternal prenatal and postpartum vitamin D3 doses selected for the trial.**
Additional file 2:
**Overview of study activities scheduled for each participant enrolled in the trial.**

## Abbreviations

ARI: acute respiratory infection
Ca:Cr: calcium-creatinine ratio
CI: confidence interval
DSMB: Data and Safety Monitoring Board
EDTA: ethylenediaminetetraacetic acid
ERC: Ethical Review Committee
FGF: fibroblast growth factor
GA: gestational age
Hb: hemoglobin
HC: head circumference
HCAZ: head circumference for age z-score
ICDDR: International Centre for Diarrhoeal Disease Research
IGF: insulin-like growth factor
IGFBP: insulin-like growth factor-binding protein
IL: interleukin
IOM: Institute of Medicine
ITT: intention to treat
25(OH)D: 25-hydroxyvitamin D
RKL: rump-to-knee length
LAZ: length for age z-score
LMP: last menstrual period
MCHTI: Maternal and Child Health Training Institute
MDIG: Maternal Vitamin D for Infant Growth
MUAC: mid-upper arm circumference
POC: point of care
PTH: parathyroid hormone
PTHrP: parathyroid hormone–related protein
SGA: small for gestational age
TIPT: Toronto Institute of Pharmaceutical Technology
TNF: tumor necrosis factor
TSC: trial steering committee
UAL: upper arm length
WAZ: Weight for age z-score
WFLZ: weight for length z-score
WHO: World Health Organization
WHO-GS: World Health Organization growth standards
