# Supplementary material for: Maternal vitamin D supplementation during pregnancy and lactation to promote infant growth in Dhaka, Bangladesh (MDIG trial): study protocol for a randomized controlled trial
Source: Trials. 2015 Jul 14;16:300. doi: 10.1186/s13063-015-0825-8 (PMC4499946; doi:10.1186/s13063-015-0825-8)
Supplement: Additional file 1: — Summary of the rationale for the maternal prenatal and postpartum vitamin D3 doses selected for the trial. [file 13063_2015_825_MOESM1_ESM.pdf]

Roth et al. Maternal vitamin D for infant growth (MDIG): study protocol for a randomized controlled trial of vitamin D supplementation during pregnancy and lactation in Dhaka, Bangladesh

Supplementary Table. Summary of the rationale for the maternal prenatal and postpartum vitamin D3 doses selected for the Maternal Vitamin D for Infant Growth (MDIG) trial in Dhaka, Bangladesh

Regimen	Explanation/Justification
Placebo	The current standard of antenatal and postpartum care in Bangladesh and most low-income countries does not include vitamin D supplementation. Moreover, a Cochrane Collaboration systematic review published in 2012 found a lack of evidence to support routine vitamin D supplementation during pregnancy[1]. Current World Health Organization guidelines do not recommend routine vitamin D supplementation[2]. The inclusion of the placebo group in the proposed trial does not place participants at risk and will enable findings to have relevant policy implications.
4,200 IU/week (prenatal)	This dose is approximately equivalent to the North American recommended dietary allowance (RDA), 600 IU/day. Dietary guidelines released in November 2010 by the US Institute of Medicine (IOM) set the RDA for vitamin D for Canadian and American pregnant and lactating women at the same level as non-pregnant adults (600 IU/day)[3]. The RDA was established to promote bone health in adults, and assumes inputs from a variety of sources in the setting of minimal sun exposure, rather than implying that 600 IU/day is a recommended supplementation dose[4].
16,800 IU/week (prenatal)	To attain vitamin D sufficiency in the majority of women, according to the IOM-recommended threshold of 50 nmol/L, we estimated that a dose equivalent to 2,000 to 2,500 IU/day would be required, based on previous pharmacokinetic studies[5, 6]. The 2,400 IU/day dose (administered as 16,800 IU/week) was selected in this range as it is a multiple of the RDA of 600 IU/day. Although some authorities have recommended doses less than 2000 IU/day in Canada and the US, such doses would be less likely to yield the 50 nmol/L steady-state in the majority of women in Dhaka, where baseline vitamin D status is lower than in typical women in North America.

Regimen	Explanation/Justification
28,000 IU/week (prenatal)	This dose is proposed to safely elevate 25(OH)D and ensure suppression of PTH secretion in most pregnant women. In a previous trial, we observed that 35,000 IU/week (~5000 IU/day) potently suppressed maternal PTH production during pregnancy in Dhaka[6]; however, earlier data suggested that 2,000 IU/day may have a weaker effect on PTH[5]. Based on the association between attained 25(OH)D and PTH at delivery in pregnant women in our preliminary trial, we expect that a dose of 4,000 IU/d (with an expected attained group mean 25(OH)D of ~110 nmol/L) will be sufficient for parathyroid suppression. The selection of a dose equivalent to 4000 IU/d balances desired physiological effects with safety considerations. Firstly, during our research, we found that 5000 IU/d (as 35,000 IU/week) did not provoke hypercalcemia and was not associated with any discernible adverse pregnancy outcomes[6]. A reduction of that dose by 20% will provide a wide margin of safety for a larger study population that can be less intensely monitored for hypercalcemia. This dose level has been studied and found to be safe in pregnant women in the United States[7]. A dose equivalent to 4000 IU/d does not exceed the current IOM UL[3], a conservative margin of safe intake for the general population, even if clinical/biochemical monitoring were not undertaken.
28,000 IU/week (postpartum)	Prenatal supplementation may impact infant length through enhanced fetal skeletal growth, and/or latent effects on infant growth that result from the larger vitamin D stores provided by supplemented mothers during gestation. We speculated that maintenance of the vitamin D steady-state during the period of lactation may accentuate the growth effects. There is a dose-response relationship linking maternal vitamin D status, breast milk vitamin D activity, and infant vitamin D status[8]. Vitamin D supplementation of lactating mothers with at least 2000 IU/day has been linked to significant increases in the 25(OH)D of breast-fed infants[9]. Maternal supplementation, rather than infant supplementation, may be preferred because of the possibility that the mechanism of effect of vitamin D on growth may involve the regulation of breast milk transfer of other endocrine factors (e.g., PTHrP), and may not only be related to the infant's 25(OH)D concentration. In addition, if shown to be beneficial, maternal supplementation may be more appealing because it would support the optimality of exclusive breastfeeding, in that all nutrients would be delivered to the infant via breast milk without the need for infant nutrient supplementation. To maximize analytical efficiency (i.e., maintain sufficient numbers of participants in each intervention group) given resources/feasibility, we aimed to test the postpartum effect only at the highest dose level (28,000 IU/week).

References (for Supplementary Table)

1. De-Regil LM, Palacios C, Ansary A, Kulier R, Pena-Rosas JP: **Vitamin D supplementation for women during pregnancy**. *Cochrane Database Syst Rev* 2012, **2**:CD008873.
2. World Health Organization.: **Guideline: Vitamin D supplementation in pregnant women**. Geneva: World Health Organization; 2012.
3. Ross AC, Taylor CL, Yaktine AL, Del Valle HB: **Committee to Review Dietary Reference Intakes for Vitamin D and Calcium, Institute of Medicine. Dietary Reference Intakes for Calcium and Vitamin D.** Washington, DC: The National Academies Press; 2010.
4. Ross AC, Manson JE, Abrams SA, Aloia JF, Brannon PM, Clinton SK, Durazo-Arvizu RA, Gallagher JC, Gallo RL, Jones G *et al*: **The 2011 report on dietary reference intakes for calcium and vitamin D from the Institute of Medicine: what clinicians need to know**. *J Clin Endocrinol Metab* 2011, **96**(1):53-58.
5. Roth DE, Mahmud A, Raqib R, Akhtar E, Black R, Baqui A: **Pharmacokinetics of high-dose weekly oral vitamin D3 supplementation during the third trimester of pregnancy in Dhaka, Bangladesh**. *Nutrients* 2013, **5**(3):788-810.
6. Roth DE, Mahmud AA, Raqib R, Akhtar E, Perumal N, Pezzack B, Baqui AH: **Randomized placebo-controlled trial of high-dose prenatal third-trimester vitamin D3 supplementation in Bangladesh: the AViDD trial**. *Nutrition Journal* 2013, **12**:47.
7. Hollis BW, Johnson D, Hulsey TC, Ebeling M, Wagner CL: **Vitamin D supplementation during pregnancy: Double blind, randomized clinical trial of safety and effectiveness**. *J Bone Miner Res* 2011, **26**(10):2341-2357.
8. Taylor SN, Wagner CL, Hollis BW: **Vitamin D supplementation during lactation to support infant and mother**. *J Am Coll Nutr* 2008, **27**(6):690-701.
9. Wagner CL, Hulsey TC, Fanning D, Ebeling M, Hollis BW: **High-dose vitamin D3 supplementation in a cohort of breastfeeding mothers and their infants: a 6-month follow-up pilot study.** *Breastfeed Med* 2006, **1**(2):59-70.
