# Supplementary material for: Maternal vitamin D supplementation during pregnancy and lactation to promote infant growth in Dhaka, Bangladesh (MDIG trial): study protocol for a randomized controlled trial
Source: Trials. 2015 Jul 14;16:300. doi: 10.1186/s13063-015-0825-8 (PMC4499946; doi:10.1186/s13063-015-0825-8)
Supplement: Additional file 2: — Overview of study activities scheduled for each participant enrolled in the trial. [file 13063_2015_825_MOESM2_ESM.pdf]

Additional file 2. Overview of study activities scheduled for each participant enrolled in the Maternal Vitamin D for Infant Growth (MDIG) trial in Dhaka, Bangladesh.

	**STUDY PERIOD**								
	**Enrolment**	**Allocation**	**Post-allocation**						**Close -out**
**Timing** (Months, in relation to delivery)	***-4 to -6***	***-4 to -6***	***-2.5***	***0***	***3***	***6***	***9***	***12***	***24***
**ENROLMENT**									
Eligibility screen	✓								
Informed consent	✓								
Obstetric ultrasound	✓								
Allocation		✓							
**INTERVENTIONS**									
Prenatal Supplementation		✓	✓	✓					
Postpartum Supplementation				✓	✓	✓			
**ASSESSMENTS**									
Visit frequency:	***Once***	***Weekly***					***Every 3 months*** [1]		
Maternal clinical assessment	✓		✓	✓	✓	✓			
Maternal specimen collection	✓		✓	✓	✓	✓			
Paternal anthropometry and specimen collection [2]	✓								
Birth outcome ascertainment				✓					
Cord blood/tissue & placental specimen collection				✓					
Infant Anthropometry				✓	✓	✓	✓	✓	✓
Infant morbidity assessments				✓	✓	✓	✓	✓	✓
Infant specimen collection					✓	✓		✓	✓

[1] Tri-monthly visits begin at 9 months of age and continue until completion of follow-up at 2 years of age.
[2] Paternal blood specimen collection will be used for epigenetic sub-studies; however, paternal data or specimen collection is not required for pregnant woman to join the trial, and therefore the consent process will be separate.
